# Mechanism of Apoptosis in Porcine Ovarian Granulosa Cells Triggered by T-2 Toxin

**DOI:** 10.3390/genes15050579

**Published:** 2024-05-01

**Authors:** Yige Chen, Xianrui Zheng, Ren Zhou, Huibin Zhang, Yangguang Liu, Xiaojing Hu, Zongjun Yin

**Affiliations:** Anhui Provincial Laboratory of Local Animal Genetic Resource Conservation and Bio-Breeding, College of Animal Science and Technology, Anhui Agricultural University, No. 130, West Changjiang Road, Hefei 230036, China; starry_cyg5027@stu.ahau.edu.cn (Y.C.); ahauzxr@ahau.edu.cn (X.Z.); zr511823@126.com (R.Z.); zhanghuibin1997@126.com (H.Z.); lyg236200@163.com (Y.L.); huxiaojing0211@163.com (X.H.)

**Keywords:** T-2 toxin, granulosa cells, apoptosis, sow

## Abstract

T-2 toxin (T-2), an A-type mono mycotoxin produced by various Fusarium species, disrupts DNA/RNA and protein synthesis upon entering the body, resulting in pathological conditions in various tissues/organs and posing a significant threat to human and animal health. However, the mechanisms underlying its toxicity remain unclear. With the goal of learning how T-2 affects reproduction in animals, we utilized primary porcine ovarian granulosa cells (pGCs) as a carrier in vitro and constructed concentration models for analyzing cell morphology and RNA-sequencing (RNA-seq). Our findings showed that T-2 could influence pGCs morphology, induce cell cycle arrest, and promote apoptosis in a dose-dependent manner. The results of RNA-seq analyses indicated that a total of 8216 genes exhibited significant differential expression (DEG) following T-2 treatment, of which 4812 were observed to be down-regulated and 3404 were up-regulated. The DEGs following T-2 toxin treatment of pGCs had a notable impact on many metabolic pathways such as PI3K-Akt, Ras, MAPK, and apoptosis, which in turn altered important physiological processes. Gene set enrichment analysis (GSEA) indicated that the differences in the harmful effects of T-2 might be caused by the varying control of cellular processes and the pathway responsible for steroid metabolism. These results present further insights regarding the mechanism of T-2 action on sow reproductive toxicity, enhance our understanding of T-2 reproductive toxicological effects, and lay a theoretical foundation for the judicious prevention of T-2-induced reproductive toxicity.

## 1. Introduction

Ovarian granulosa cells, somatic cells that interact with the oocyte and support oogenesis within the follicle, play a crucial role in follicular development and ovulation, necessitating proper proliferation and functional regulation [1]. Nevertheless, only a minority of follicles undergo maturation and ovulation, while most follicles decline in reproductive efficacy owing to degenerative atresia before ovulation, and apoptosis of granulosa cells is a crucial factor that instigates follicular atresia [2]. Apoptosis in ovarian granulosa cells can be attributed to a multitude of factors, broadly categorized as either endogenous or exogenous; The former category mainly consists of hormones and cytokines released by the organism, which regulate apoptotic factors and induce apoptosis; the latter includes human factors, environmental pollutants, and other similar agents [3]. T-2, one of the most toxic trichothecenes and a widely distributed contaminant in various agriculture commodities including wheat, barley, oats, and corn [4], exhibits remarkable stability at ambient temperature for extended periods and cannot be degraded to reduce its toxicity by heat, high pressure, ultraviolet radiation, as well as neutral or acidic conditions. Consequently, the stability of this chemical increases the likelihood of exposure for both humans and animals.

Previous research demonstrated that rats treated with T-2 exhibited both neurochemical injury and altered adaptive or compensatory processes simultaneously, as observed by changes in brain neuronal nuclear chromatin. In the enteric nervous system (ENS), T-2 can change the expression of cocaine- and amphetamine-regulated transcript (CART) [5]. T-2 can trigger the intrinsic apoptotic pathway by facilitating the movement of bax to the outer membrane of the mitochondria, causing a decrease in the potential across the mitochondrial membrane, opening of PTPC, and release of cytochrome c (but not AIF). In addition, at the cellular level, T-2 activates various signal transduction pathways in human neural cells, leading to apoptosis. T-2 has a significant impact on many cell membrane functions in L-6 myoblasts, even at extremely low doses ranging from 0.4 pg/mL to 4 ng/mL. Chronic exposure of pigs to subclinical doses of T-2 leads to altered immune cell polarization, a key factor in immune response regulation [6]. These findings suggest that T-2 exhibits highly toxic activity, damaging mitochondria, inhibiting protein and DNA/RNA synthesis, disrupting cell membrane structure, and causing harm to cells with high proliferative activity. Animals are most commonly exposed to T-2 through the consumption of feed that has been polluted, leading to various diseases and tissue or organ damage, including fatalities in humans and other animals [7]. For example, low concentrations of T-2 can disrupt enterocyte metabolism and evoke inflammation of the mucous membrane of the small intestine. Sows exposed to contaminated feed containing 1–2 ppm T-2 toxin during the third trimester of pregnancy experienced ovarian inhibition, leading to histopathological degeneration and associated atrophy [8]. Thus, exploring the cytotoxic effects of T-2 and its mechanism on ovarian granulosa cells will provide a better understanding of its effect on female reproductive health.

Previous studies have shown that the T-2 toxin exerts significant direct dose-dependent effects on granulosa cell steroidogenesis, affecting reproductive performance [9]. However, the potential mechanism by which the T-2 toxin triggers apoptosis in granulosa cells remains unclear. In this study, we explored the toxicological effects of T-2 on pGCs in vitro through T-2 exposure and RNA-seq analyses. These findings are expected to enhance our understanding of the cellular and molecular mechanisms of T-2 on pGCs.

## 2. Materials and Methods

### 2.1. Ethics Statement

The experiments were conducted in full adherence to the protocols approved by the Anhui Agricultural University Animal Ethics Committee under Permission No. AHAU20180615.

### 2.2. Sample Collection, pGCs Cultured In Vitro and Exposed to T-2

The pGCs isolation and culture were performed as described. In brief, fresh Landrace ovaries (*n* > 50, to minimize potential individual animal variations), approximately 180 days old, weighing around 110–120 kg, were collected from a local commercial abattoir (Hefei, Anhui, China) for this experiment. These ovaries exhibited smooth surfaces, were healthy in development, lacked corpus luteum, and contained a substantial number of luminal follicles that were evenly distributed and fully developed. Following slaughter, the ovaries were promptly gathered and rid of nearby adipose, oviduct, and bursa tissue. Subsequently, they were submerged in a sterile saline solution at a temperature of 37 °C, which included a 1% mixture of penicillin/streptomycin (WISENT, Nanjing, China). The individuals were expeditiously conveyed to the laboratory within a thermos flask. Following repeated rinses with phosphate-buffered saline (PBS, pH 7.4) (Beyotime, Shanghai, China) at 37 °C, avoiding blood vessels, the ovaries were aspirated using a disposable sterile syringe (1 mL) inserted shallowly into the antral follicles (n > 200, measuring 3–6 mm in diameter) to collect the follicular fluid. Afterwards, the fluid was cleansed, subjected to centrifugation (1000× *g*, 5 min), reconstituted, and centrifuged once more, resulting in the collection of pGCs. The cells were grown at a temperature of 37 °C with 5% CO_2_ in Dulbecco’s modified Eagle medium/Nutrient Mixture F-12 medium (DMEM/F12, Gibco, Grand Island, NY, USA) supplemented with 10% fetal bovine serum (FBS) (Gibco, Carlsbad, CA, USA), 100 units/mL penicillin, and 100 mg/mL streptomycin (Gibco, Carlsbad, CA, USA) after being placed in a 60 mm cell culture dish (Corning, 430166, Somerville, MA, USA).

Highly purified T-2 was procured from Macklin Technology Co., Ltd. (CAS number: 21259-20-1, Shanghai, China), with a purity exceeding 99.5%. T-2 was solubilized in DMSO (Sigma-Aldrich, St. Louis, MO, USA) to create a concentrated solution of 100 μmol/L, which was then stored in a dark environment at a temperature of −20 °C for future use. The primary pGCs could be cultivated until they reached 80% confluence (when they completely covered the surface of the cell culture dish, typically after 1 week). Afterwards, the pGCs were placed in a 12-well plate (Corning, 430166, Corning, NY, USA) at a concentration of 8~10 × 10^5^ cells per well, as determined by cell counting plates (Watson, 177-122c, Kobe, Japan), and given 12 h to adhere. The purity of pGCs (with a purity level exceeding 90%) was assessed using flow cytometry. Then, the cells were exposed to various dosages of T-2 (0 nmol/L, 30 nmol/L, 60 nmol/L, and 90 nmol/L) for 24 h, while the control group received an equivalent amount of culture medium (at least three biological replicates for both treatment and control groups).

### 2.3. Cell Morphological Assay

Following exposure of pGCs to varying concentrations of T-2 (0 nmol/L, 30 nmol/L, 60 nmol/L, and 90 nmol/L) for 24 h, the compared images were obtained by the utilization of a microscope (Leica Microsystems DM2500, Wetzlar, Hessen, Germany).

### 2.4. Cell Apoptosis Analysis

T-2 was applied to the pGCs in doses of 0 nmol/L, 30 nmol/L, 60 nmol/L, and 90 nmol/L for 24 h. Afterward, the pGCs were gathered, preprocessed, subjected to trypsinization for a duration of 1 min, centrifuged (1000× *g*, 3 min), rinsed twice with PBS, and then resuspended in 500 µL of binding buffer containing 5 µL of Annexin V-fluorescein isothiocyanate (FITC) and 10 µL of propidium iodide (PI) (Bestbio, Shanghai, China). The sample acquisition was carried out using a FACS Calibur^TM^ flow cytometry instrument (FACSCalibur, BD, Franklin Lakes, NJ, USA), and the data were analyzed using FlowJo software (v7.6.1, Stanford University, Stanford, CA, USA).

### 2.5. Cell Cycle Detection

After exposure of pGCs for 24 h to T-2 at various doses (0 nmol/L, 30 nmol/L, 60 nmol/L and 90 nmol/L), the cells were gathered, fixed in 70% cold ethanol at 4 °C for 2 h, and preserved overnight at a temperature of −20 °C. After twice being cleaned with PBS, the fixed cells were exposed to 100 μL of Ribonuclease A (RNase A) (Keygen, Nanjing, China) at 37 °C for 30 min, followed by 400 μL of PI staining at 4 °C for 30 min in the dark. Cell cycle profiles were immediately detected by a flow cytometry instrument, and the cell cycle data were analyzed by using ModFit LT Software (v3.0, Variety Software House, Inc., Topsham, ME, USA).

### 2.6. Phalloidin Staining

The pGCs were placed in a 12-well plate for phalloidin staining and left to attach for 12 h. The cells were subsequently exposed to T-2 medium at concentrations of 0 nmol/L, 30 nmol/L, 60 nmol/L, and 90 nmol/L and incubated at a temperature of 37 °C for 24 h. Thereafter, antifade with fluorescein isothiocyanate (FITC) was added to the cells and darkly incubated at 20 °C for 45 min, followed by PBS wash for 8~10 min. FITC-Phalloidin (CA1620, Solarbio, Beijing, China) was applied to re-stain the sample and incubated for 30 min, after which 10 μL of an anti-fluorescence quenching solution containing 4′,6-diamidino 2-phenylindole (DAPI) was added to obtain a cytoskeleton labeled with FITC-Phalloidin and nuclei labeled with DAPI. Ultimately, the cells were scrutinized using a fluorescence microscope (Leica Microsystems DM2500, Wetzlar, Hessen, Germany).

### 2.7. Total RNA Extraction, Library Construction and Sequencing

pGCs were placed in a 55 cm^2^ dish for cell culture and incubated in DMEM/F12 medium with or without T-2 (60 nmol/L) for a duration of 24 h. As previously indicated in our report, the total RNA of both the treatment and control groups was extracted. RNA was extracted using the RNeasy Mini Kit (Qiagen, Hilden, Germany) following the manufacturer’s instructions and transferred to Majorbio (Shanghai, China) for further analysis. The assessment of the total RNA concentration, purity, and integrity was conducted through the utilization of NanoDrop 2000 (Thermo Fisher Scientific, Waltham, MA, USA) and Agilent Bioanalyzer 2100 systems (Agilent Technologies, Santa Clara, CA, USA). The quality of the RNA was proven to be sufficient for sequencing requirements, as shown in Table S1. All samples were subjected to paired-end sequencing on an Illumina Hiseq.4000 platform (Illumina, San Diego, CA, USA) [10]. The raw data, which were in fastq format, were initially processed using custom perl programs. During this stage, the data were purified by eliminating reads that contained adapters, poly-N sequences, and low-quality reads. A low-quality read is defined as a read in which over 50% of the bases have a quality value (Q-score) of ≤20 in the raw data. The subsequent studies were conducted using data that were of superior quality and free from any impurities. The reference genome and annotated file were acquired from the Ensembl database (http://www.ensembl.org/, Sus scrofa v11.1, accessed on 30 October 2023). The fragments per kilobase of exon per million reads (FPKM) value was used to quantify the gene expression levels, and HTSeq (v0.6.1) was used to count the number of reads mapped to each gene [11]. Differentially expressed genes (DEGs) were identified using the DESeq R software (v3.6.3) that provided statistical routines for determining the differential expression in digital gene expression data. The fold change |log_2_FC| and the FDR (False Discovery Rate, FDR is the multiple hypothesis testing corrected *p*-value) values were both utilized to determine if a DEG was significant or not. Statistical approaches in digital gene expression data analysis utilize a model based on the negative binomial distribution to discover differential expression. The resulting *p*-values were adjusted using Benjamini and Hochberg’s method to lower the rate of false discovery; a statistically significant result was determined as an adjusted *p*-value of 0.05 or less.

The pathways, including the significantly enriched genes, were identified using the Kyoto Encyclopedia of Genes and Genomes (KEGG, http://www.genome.jp/kegg, accessed on 28 December 2023) and Gene Ontology (GO, http://www.geneontology.org/, accessed on 12 December 2023) analyses. Gene set enrichment analysis (GSEA, https://www.gs ea-msigdb.org/gsea/index.jsp, accessed on 10 January 2024) was performed to further gain insights at the gene-set level and confirm the functional analysis results [12]. We used the signal-to-noise normalization approach to rank the genes after supplying a gene expression matrix, and filters for gene set size were set at 15 at the least and 500 at the maximum.

### 2.8. Real-Time Quantitative PCR

In order to validate the accuracy of RNA-sequencing, real-time quantitative PCR (RT-qPCR) was conducted. A random selection of twelve genes was made to determine the expression trend of the sequencing findings. Total RNA from the cells was extracted using the TRIzol reagent (Invitrogen Corporation, Carlsbad, CA, USA), and the extracted total RNA was reverse-transcribed into cDNA employing the Primer-Script RT Reagent Kit (TaKara, Tokyo, Japan). The RT-qPCR analysis was conducted using the CFX96 Touch Real-Time PCR Detection System (Bio-Rad, Hercules, CA, USA). Based on sequence information from the NCBI database (www.ncbi.nlm.nih.gov/genbank, accessed on 29 January 2024), Primer Premier 6.0 software (Premier Biosoft International, Palo Alto, CA, USA) [13] was used to design and modify the precise primers used for RT-qPCR, which are listed in Table 1. The thermal cycling protocol comprised an initial step at 95 °C for 30 s, followed by 40 cycles at 95 °C for 5 s and 60 °C for 30 s. The gene expression levels were normalized to those of *GAPDH* (glyceraldehyde-3-phosphate dehydrogenase, housekeeping gene), and the relative gene expression changes were calculated using the 2^−ΔΔCt^ method [14]. Each gene was represented as the mean ± standard deviation (SD) from three biological replicates.

### 2.9. Statistical Analysis

Every experiment was carried out three times, and the results were shown as the mean ± SD. We employed a one-way ANOVA to ascertain the statistical significance using the GraphPad Prism software (v9.0.0, GraphPad Inc., La Jolla, CA, USA). A *p*-value less than 0.05 was regarded as statistically significant; a *p*-value less than 0.01 was considered very significant; and a *p*-value less than 0.001 was considered extremely significant.

## 3. Results

### 3.1. Effect of T-2 on pGCs Morphology Changes

**Figure 1:**
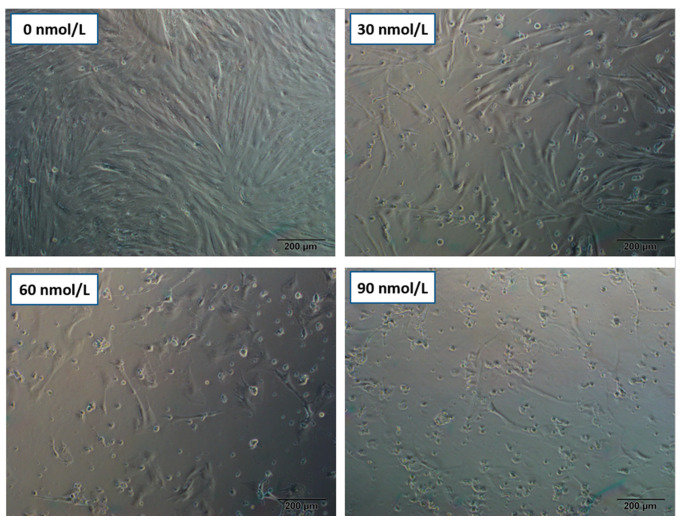
Cellular morphological observation from microscopy. Porcine ovarian granulosa cells (pGCs) were exposed to T-2 at different doses (0 nmol/L, 30 nmol/L, 60 nmol/L, and 90 nmol/L) for 24 h. Scale bar 200 μm.

In order to examine the cytotoxic impact of T-2 on cellular morphology, pGCs were treated with increasing concentrations (0 nmol/L, 30 nmol/L, 60 nmol/L, and 90 nmol/L) of T-2 for 24 h. Several visual field areas (*n* > 5) were selected at random, and the cell morphology observations were obtained as depicted in Figure 1. The pGCs belonging to the control group (0 nmol/L, 24 h) grew healthily, with fusiform shape, complete extension, compact arrangement, translucency, and sufficient adhesion.

In contrast, the T-2-treated groups demonstrated smaller granulosa cells and darker cytoplasm in color. As the T-2 concentration increased, the cells also displayed dendritic junctions. At a high dose of T-2 (90 nmol/L, 24 h), pGCs grew spherically, and a significant proportion of cells demonstrated shedding, suspension, dehiscence, and apoptosis.

**Figure 2:**
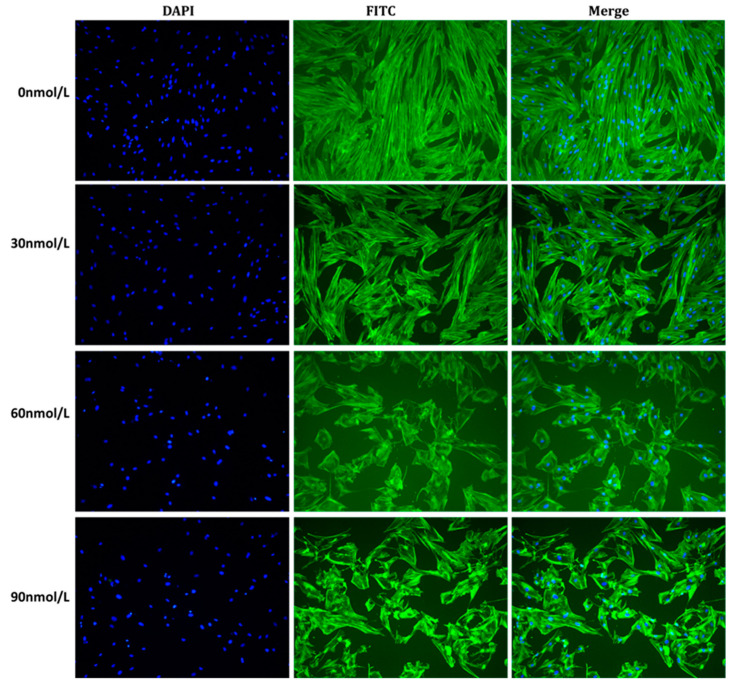
Fluorescence microscopy image of the cytoskeleton morphology. The granulosa cells of the swine ovary were seen using laser scanning confocal microscopy. The fluorescent immunocytochemistry technique was employed to stain cells with varying concentrations of T-2 (0 nmol/L, 30 nmol/L, 60 nmol/L, and 90 nmol/L) for a duration of 24 h. Actin skeletons were stained with FITC-phalloidin, resulting in a green coloration, while cell nuclei were stained with DAPI, resulting in a blue coloration. Scale bar 200 μm.

The morphological changes in cytoskeleton structure in pGCs are shown in Figure 2. In the control group (0 nmol/L, 24 h), granulosa cells exhibited normal morphology, characterized by distinct bundles of actin fibers. Treatment with T-2 resulted in a noticeable reduction in the number of actin fiber bundles. The actin cytoskeleton collapsed and shrank with increasing T-2 concentrations, indicating a lack of organized cytoskeleton and tensile fiber. Furthermore, in both treatment groups of 60 nmol/L and 90 nmol/L, the cell boundaries were unclear, and bundles of actin fibers were lost in cells.

### 3.2. Effect of T-2 on pGCs Apoptosis

**Figure 3:**
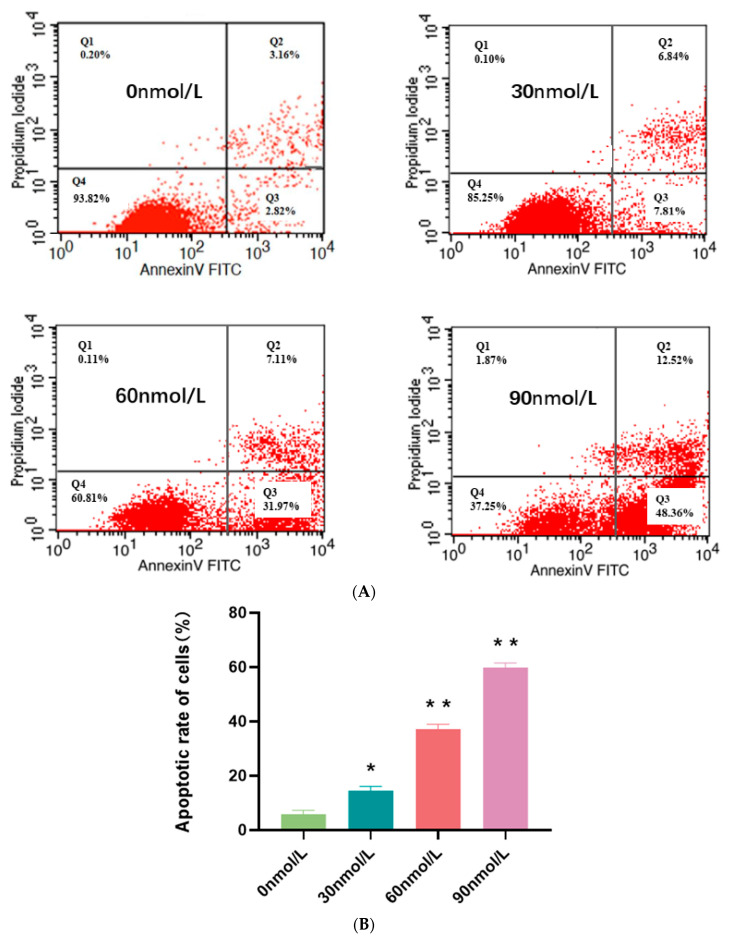
Dose-dependent effects of T-2 on apoptosis in pGCs. (**A**) Impacts of various T-2 concentrations (0 nmol/L, 30 nmol/L, 60 nmol/L, and 90 nmol/L) for 24 h on pGC apoptosis by flow cytometry after Annexin V-FITC and PI staining. One representative of the three independent experiments was displayed. (**B**) The calculation of apoptotic cell percentage. Compared to the control group, n = 3, * *p* < 0.05, ** *p* < 0.01. All experiments were repeated three times.

We employed flow cytometry in conjunction with Annexin V-FITC/PI double staining to detect whether T-2 could induce apoptosis in pGCs, and the levels of T-2-induced apoptosis are displayed in Figure 3. The apoptotic cell population gradually increased after cells were exposed to T-2 (30~90 nmol/L) for 24 h, and the 30 nmol/L (24 h) treatment group showed a considerably higher apoptosis rate than that in the control (0 nmol/L, 24 h) (*p* < 0.05). When the T-2 concentrations were between 60 nmol/L (24 h) and 90 nmol/L (24 h), the apoptotic rate differences were much more noticeable (*p* < 0.01). These findings indicated that T-2 exhibited a dose-dependent impact on inducing apoptosis in pGCs.

### 3.3. T-2 Induced Cell Cycle Alteration

**Figure 4:**
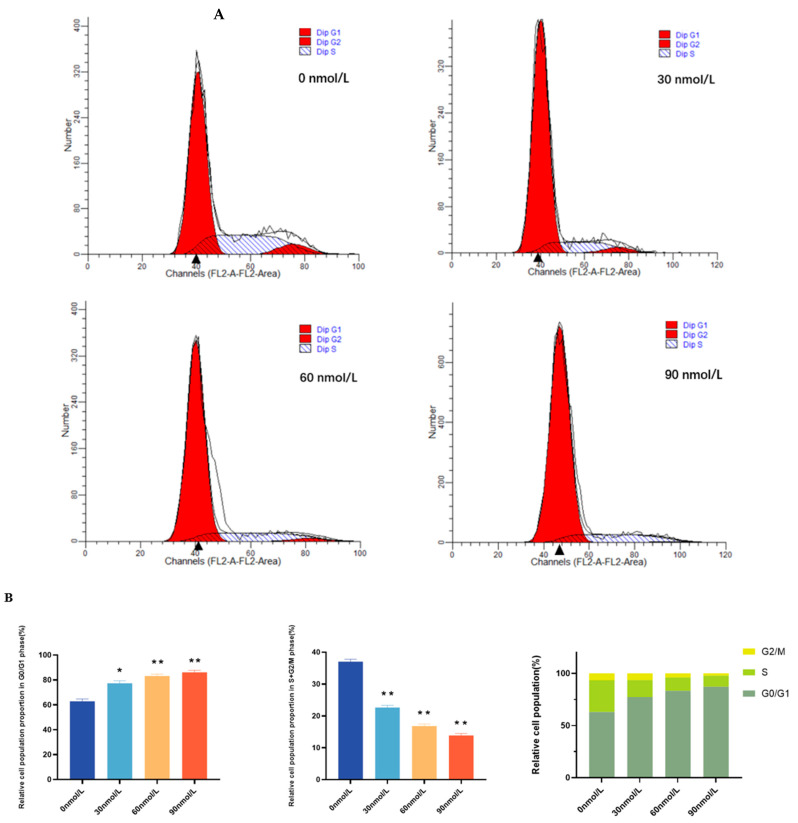
Cell cycle arrest induced by T-2 in pGCs. (**A**) pGCs cells were exposed to various concentrations of T-2 (0 nmol/L, 30 nmol/L, 60 nmol/L, and 90 nmol/L) for 24 h and flow cytometry was used to assess the distribution of the cell cycle. A single sample from each of the three separate experiments was presented. (**B**) Distribution of relative cell population proportion of pGCs in cell cycle phases after treatment with different concentrations of T-2 (0 nmol/L, 30 nmol/L, 60 nmol/L, and 90 nmol/L) for 24 h. Both * and ** represented significant differences at *p* < 0.05 versus control and *p* < 0.01 versus control, respectively. The experiments were replicated three times and were conducted independently on three occasions.

To investigate the effect of T-2 on the cell cycle and its regulation, the control (0 nmol/L, 24 h) and toxin-treated cells (30 nmol/L, 60 nmol/L, and 90 nmol/L for 24 h, respectively) were stained with PI and examined by flow cytometry. Analysis of cell cycle profiles showed that T-2 treatment induced a marked accumulation of cells during the G0/G1 cell cycle phase in a dose-dependent manner with increasing T-2 concentration. Meanwhile, the T-2-treated groups (30 nmol/L~90 nmol/L) were shown to exhibit a highly significant and consistently reduced proportion of S and G2/M phase cells compared to the control group (*p* < 0.01) (Figure 4). In general, we found that the cell cycle indicated that T-2 inhibited cell cycle progression, which led to the G0/G1 phase arrest of pGCs.

An optimum dose of 60 nmol/L T-2 toxin for 24 h was chosen for further analysis, which was based on the findings from studies on cell cycle, cell apoptosis, and cell morphology. Specifically, as the T-2 concentration increased (24 h), pGCs exhibited a size reduction accompanied by cytoskeletal crinkling and deformation. The pGCs in the T-2-exposure groups (60 nmol/L, 24 h) showed extremely significant levels of change in apoptosis rate and G0/G1 phase cell number ratio (*p* < 0.01). Thus, we selected 0 nmol/L and 60 nmol/L for the subsequent RNA-seq studies, each with three biological replicates.

### 3.4. Sequencing Analysis of Gene Expression

**Figure 5:**
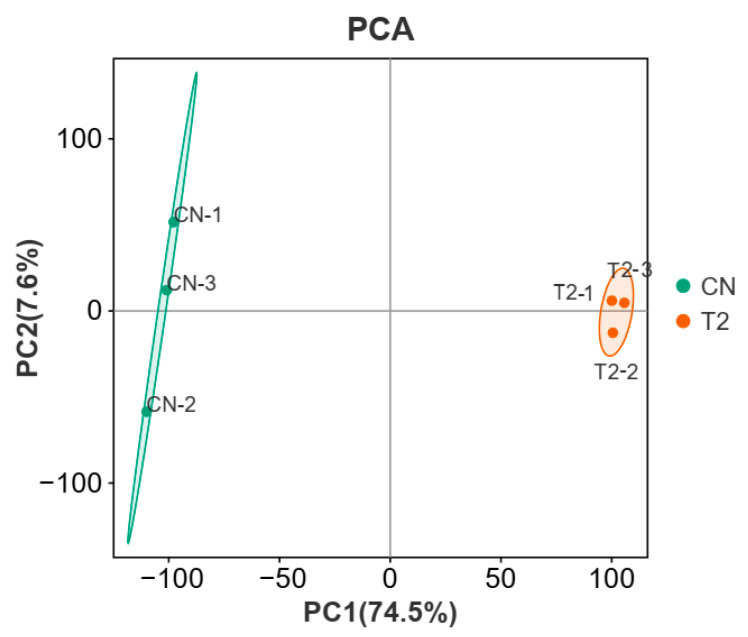
Principal component analysis of samples relationships. The experimental groups (T2-1,2,3) were denoted by orange circular nodes, while the control groups (CN-1,2,3) were denoted by green circular nodes. PC1 and PC2, the two main components, collectively explained 82.1% of the overall variance. PC1 accounted for 74.5% of the variance, while PC2 accounted for 7.6%. The light green and orange shading represented the population clustering and confidence ellipses for the control and treatment groups, respectively.

From the sequencing results, a total of 8,546,605,500 and 11,777,073,900 bp raw reads were generated in each group. The obtained sequencing data successfully met all quality control requirements, with a Q20 score (base sequencing error probability < 1%) exceeding 97% and a Q30 score (base sequencing error probability < 0.1%) exceeding 94% in each group (Table S2). Principal component analysis (PCA) was conducted on the gene expression patterns of 6 samples, and the relationships between samples were visualized on the first two main components. The two primary components accounted for 82.1% of the total variation. PC1 accounted for 74.5% of the variance, while PC2 accounted for 7.6% of the variance. The results indicated that the sequencing quality met the necessary criteria for subsequent analysis, and the gene expression of three biological replicates exhibited a consistent pattern (Figure 5). Following these rigorous quality control steps, the data underwent downstream analysis. The NCBI SRA database contains all read data (project ID: SRP254496).

### 3.5. Analysis of Differentially Expressed Genes

**Figure 6:**
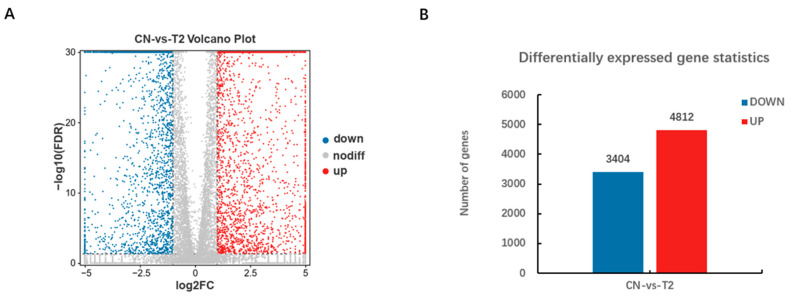
The differentially expressed genes (DEGs) identification. (**A**) DEGs with up- and down-regulated expression levels were seen in the volcano plot. Genes with a fold change|log2FC| ≥ 1 and an FDR ≤ 0.05 were shown as blue and red dots, respectively, indicating substantial differences. Grey points, signifying non-significant differences, were used to represent genes with fold changes |log2FC| < 1 and FDR < 0.05. Up-regulated and down-regulated DEGs were indicated by red and blue dots, respectively. (**B**) Up- and down-regulated expression levels of DEGs statistics. The number of DEGs was represented on the Y-axis, while up-regulated and down-regulated DEGs were represented on the X-axis.

**Figure 7:**
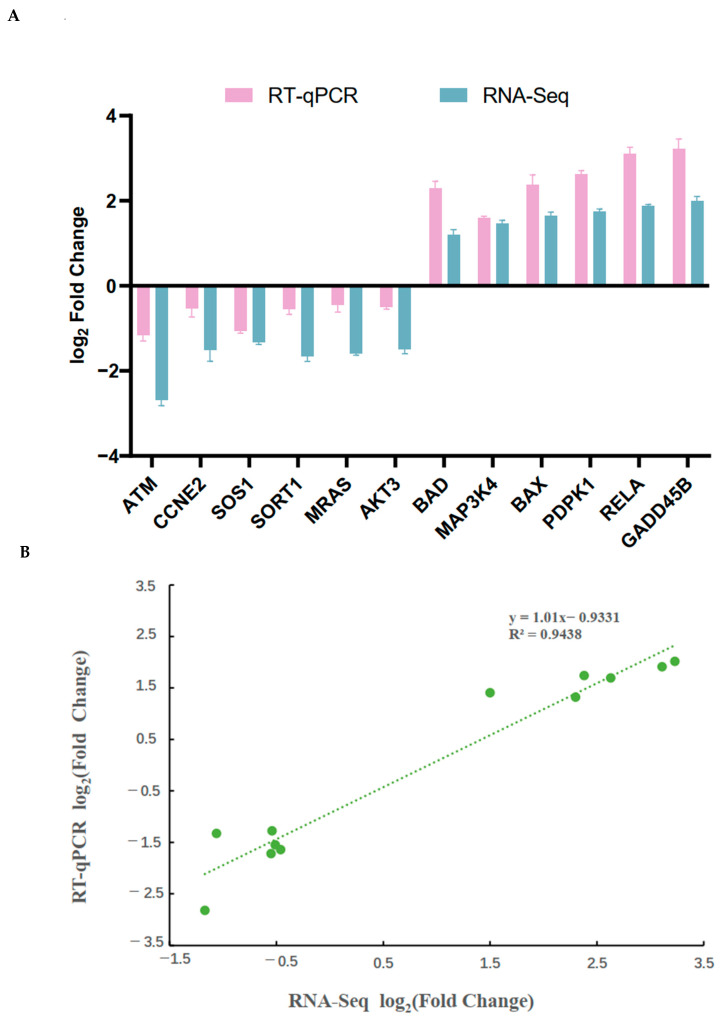
Validation of DEGs identified from RNA-seq data between control and treatment groups (T-2 60 nmol/L, 24 h) using real-time quantitative PCR (RT-qPCR). (**A**) A comparison of validation data from RT-qPCR and RNA-seq. The Y-axis represented the Log2 ratio for the control and T-2 (60 nmol/L, 24 h) treatment groups; the X-axis represented DEGs in the validated comparison of this study. (**B**) The graphic displayed the linear regression model, R-squared value, and expression difference of the 12 selected DEGs between the treatment groups (60 nmol/L, 24 h) and the control groups (0 nmol/L, 24 h). The green points represented these DEGs. The Y-axis and the X-axis corresponded to the log2 ratio for RT-qPCR and RNA-seq, respectively. The correlation trend was depicted by a green dotted line. All experiments were replicated thrice.

The RNA-seq results revealed the expression of 22,342 genes after alignment to the reference genome Scrofa 11.1. 8216 genes were found to be significantly differentially expressed based on the criteria of a fold change |log_2_FC| ≥ 1 with FDR ≤ 0.05 for two different treatment comparisons of pGCs (Figure 6A and Table S3); a fold change |log_2_FC|< 1 with FDR > 0.05 was considered non-significant. Of these, 4812 were up-regulated, while 3404 were down-regulated (Figure 6B). A total of twelve genes were randomly chosen for verification through RT-qPCR to verify the dependability of the sequencing data, six (*ATM, CCNE2, SOS1, SORT1, MRAS,* and *AKT*) were found to be significantly down-regulated in the treatment group (T-2 60 nmol/L, 24 h), and the other six genes (*BAD, MAP3K4, BAX, PDPK1, RELA,* and *GADD45B*) were observed to be significantly up-regulated. The consistency between the sequencing and RT-qPCR results demonstrated the validity of the RNA-seq data (Figure 7).

### 3.6. RNA-Seq Data Bioinformatic Analysis

**Figure 8:**
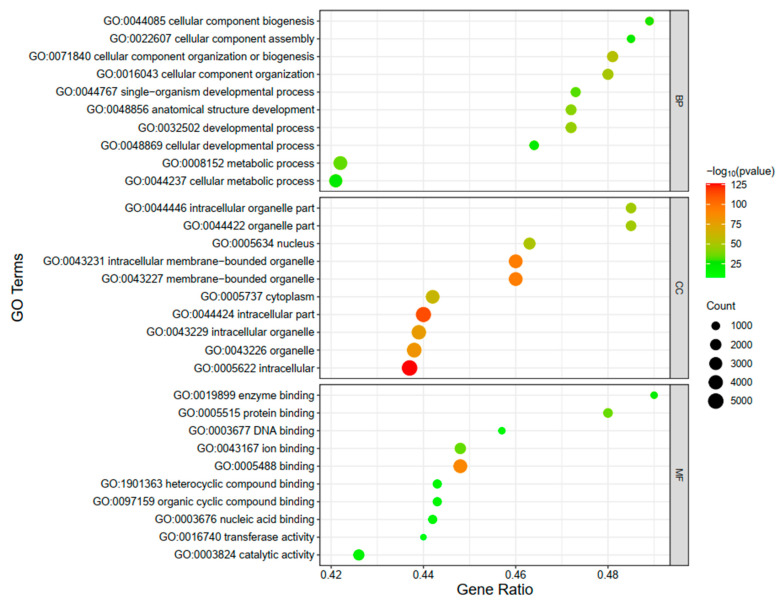
GO enrichment analysis and classification statistics from the DEGs. The ordinate represented the top 10 GO terms based on three major categories (BP, CC, and MF), and the abscissa represented the gene ratio; gene ratio: the number of DEGs enrichment in this GO term/the total number of DEG used for enrichment analysis. As the value increased, the level of enrichment also increased. The color gradient, ranging from red to green, visually illustrated the trend of *p*-values from low to high; an adjusted *p*-value < 0.05 was considered significant in GO analysis. The bubble size represented the DEGs count number.

**Figure 9:**
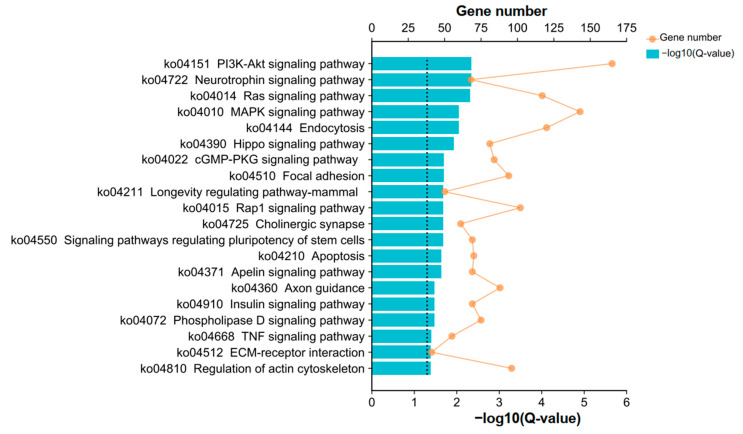
The top 20 significantly enriched KEGG pathways from DEGs. The X-axis at the top and bottom indicated the number of genes corresponding to points on the broken line and the significance of enrichment corresponding to the bar’s height, respectively. The Q-value of 0.05 was the significant enrichment pathways critical value, which was represented by black dotted lines.

To gain a more comprehensive understanding of the identified DEGs, we performed KEGG pathway and GO enrichment analyses for DEGs. GO terms categorization associated with 8216 DEGs was performed based on three main categories, namely biological process (BP), cellular component (CC), and molecular function (MF). The findings resulting from the enriched analysis indicated that a total of 108, 51, and 38 GO terms were found to be significantly enriched in the BP, CC, and MF categories (adjusted *p*-value < 0.05) (Table S4), respectively. Biological process enrichment analysis revealed that cellular component organization or biogenesis, cellular component organization, developmental process, and cellular metabolic process were the most enriched. Moreover, most of the significantly enriched molecular function-related GO terms were associated with activity and binding. The cellular components with the highest enrichment of DEGs were mainly associated with the nucleus, cytoplasm, intracellular region, organelle, and membrane. The top 10 enriched GO terms for each category are shown in Figure 8, and it could be inferred that the biological functions that these DEGs were mainly focused on during T-2-induced pGCs apoptosis were linked to changes in cellular components and activities. According to KEGG enrichment analysis (Figure 9 and Table S5), the PI3K-Akt signaling pathway, which was involved in various processes such as the recruitment of primordial follicles in the ovary, proliferation of granulosa cells (GC), luteinization, and maturation of oocytes [15], was found to be the most significantly enriched among the top 20 essential pathways (Q-value < 0.05; Q-value = *p*-value corrected for multiple hypothesis testing). In addition, the Ras and MAPK signaling pathways were enriched in an adjacent manner, of which activation or inhibition could effectively regulate the expression of mitotic process proteins in chicken ovarian granulosa cells [16]. Finally, multiple cell processing processes, including “Apoptosis”, “Signaling pathways regulating pluripotency of stem cells” and “Ras signaling pathway”, were enriched, which indicated that pGCs after exposure to T-2 might be involved in many biological processes such as cell growth, apoptosis, differentiation, and metabolism.

**Figure 10:**
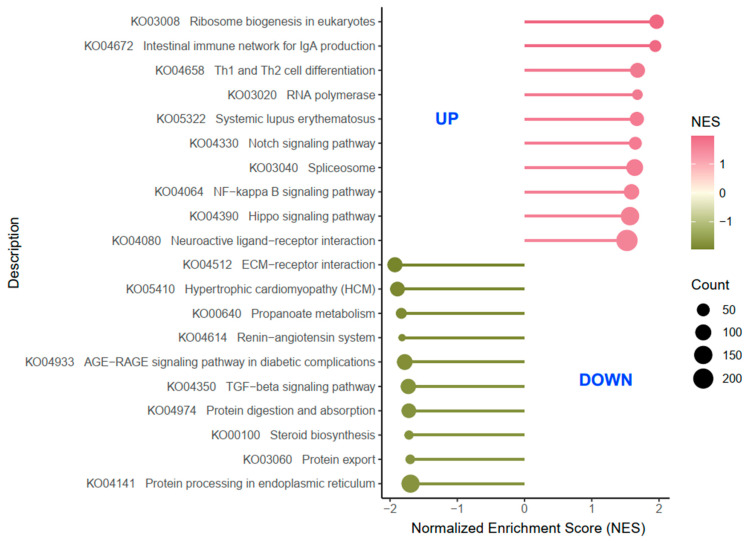
Top 10 up-regulated and down-regulated enriched hallmark signaling pathways by GSEA in pGCs with T-2-exposed compared to controls (CN). NES: normalized enrichment scores. The green-to-pink color gradient represents the NES trend from low to high. NOM *p*-value < 0.05, FDR q-value < 0.25, and |NES|>1 were considered significant in GSEA. The bubble size represented the genes count number.

To avoid the limitations of DEG enrichment analysis and the inability to determine the overall expressions of some important pathways related to pathology and physiology after T-2 administration, we performed GSEA to gain a comprehensive insight into the potential mechanisms of T-2-induced pGCs damage. GSEA showed that the TGF-β signaling pathway and steroid biosynthesis were significantly impaired in T-2-treated pGCs, with normalized enrichment scores (NES) all less than −1.70 and false discovery rates (FDR) of around 0.05 (NOM *p*-value < 0.05, FDR q-value < 0.25, and |NES| > 1) (Figure 10 and Figure S1). In contrast, Notch, NF-kappa B, and Hippo pathways were significantly up-regulated in T-2-treated pGCs, with NES and FDR ranging from 1.5681 to 1.6482 and from 0.0938 to 0.1552, respectively. These results revealed that exposure to T-2 would directly cause activation or inhibition of related cellular signaling pathways, ultimately leading to apoptosis in pGCs.

## 4. Discussion

Granulosa cells are a significant category of ovarian cells situated external to the zona pellucida that play important roles in regulating follicular atresia besides several other ovarian functions [17]. Granulosa cell apoptosis can lead to follicular atresia and inhibit oocyte development, which can adversely affect reproductive efficiency. The apoptosis of granulosa cells is regulated by various factors such as hormones and cytokines, involving changes in multiple signaling pathways [18]. It is widely recognized that granulosa cells (GCs) can easily undergo luteinization when grown for a long time in the serum culture system in vitro, which serves as a useful model for both luteinization and follicular atresia. Prior studies have demonstrated that the molecular process occurring during the luteinization of granulosa cells involves a transition from producing estrogens to producing progestogens. Morphologically, this transition is characterized by the transformation of spherical granulosa cells into spindle (star)-shaped luteinized cells with numerous extensions [19]. In this work, pGCs refers to luteinized granulosa cells, which were identified based on cell shape and culture duration. However, their capacity to release sex hormones was not assessed. T-2 toxin is a widely studied trichothecene toxin that can cause extensive toxicity to species under different exposure pathways [20]. Balb/c mice (4-week-old) treated intraperitoneally with T-2 (1.75 mg/kg bw) or saline vehicle, when challenged with reovirus, showed increased lung viral burden, bronchopneumonia, and pulmonary cellular infiltration [21]. Earlier studies have shown that its female reproductive toxicity was manifested by impairing female reproductive function and reducing fertility [22]. Pregnant rats on day 13 of gestation were treated orally with 2 mg/kg of T-2, resulting in oxidative stress followed by the activation of the MAPK pathway, triggering apoptosis in the fetal brain and the mother. Pregnant mice exposed to T-2 showed disrupted liver glycolipid metabolism in the young mice. The combination of T-2 and HT-2 toxins with IGF-I had the ability to modify the secretion of progesterone by porcine ovarian cells cultivated in vitro and perhaps regulate the production of ovarian steroids [23].

In this study, we have shown that treating pGCs with T-2 induces morphological alterations, apoptosis, and cell cycle arrest. Apoptosis is common in various biological processes such as immune response, embryonic development, normal tissue, and organ degeneration, of which morphological changes are relatively intuitive, including cytoskeleton disintegration, chromatin condensation, and cell shrinkage. Numerous previous studies have shown that T-2 toxin can induce apoptosis in a variety of cells with high proliferative activity. Quiroga et al. [24] documented the ultrastructural alterations observed in thymocytes, indicating the presence of apoptosis in mice that were administered T-2 toxin. Subsequently, Shinozuka et al. [25] provided further elucidation from several perspectives and affirmed that the lymphoid cell death triggered by the T-2 toxin was indeed apoptosis, which presented the initial documentation of apoptosis induced by mycotoxins. T-2 triggers apoptosis through the production of reactive oxygen species, which facilitates the movement of cytochrome c from the mitochondria to the cytoplasm. This, in turn, promotes the creation of apoptosomes. Cell cycle arrest is also one of the effects of T-2-induced cytotoxicity. Fatima et al. [26] have demonstrated that the T-2 toxin causes early and late apoptosis and induces G0/G1 phase cell cycle arrest in rat cells by altering cyclin D1 mRNA and the Bax/Bcl-2 ratio, which are consistent with our findings. Additionally, T-2 also has cytotoxic and immunosuppressive properties, and it is a strong inhibitor of mitochondrial activity and protein synthesis both in vivo and *in vitro*. T2-toxin could interfere with DNA and protein synthesis in the bone marrow, spleen, and thymus [27]. The underlying mechanism by which T-2 causes DNA damage and thus induces apoptosis is oxidative stress [28]. A study conducted in live mice showed that a single dose of T-2 improves the immunological response in the spleen, accompanied by an increased expression of miR-155-5p [29]. T-2-induced cytotoxicity in MOLT-4 and IM-9 cells is caused by the induction of early apoptosis and cell membrane damage, respectively. The co-occurrence of T-2 and its metabolites can pose a potential threat to reproductive health due to antagonistic interactions [30]. The findings of our present investigation were in line with previous data on alterations in cell morphology and the harmful impacts of T-2 on cells.

The cellular response to environmental changes is mediated by a multitude of cellular biological processes and signaling pathways that are extensively implicated in various cellular functions, which effectively receive, amplify, and integrate extracellular signals, ultimately resulting in genetic and physiological alterations. The GO enrichment analysis indicated that the DEGs were mostly associated with intracellular activities, binding events, cellular metabolic processes, cellular component organization, and other key terms. Regarding BP terms, we also found that the enriched GO functions of down-regulated and up-regulated DEGs were connected to “metabolic processes” and “response to stress” (Table S4), which indicated that pGCs responded to organic stimuli with active metabolic and cellular activities when treated with T-2. The significant enrichment of “cell cycle”, “cell morphogenesis”, and “mitotic cell cycle” corresponded with the results of our study; the “reproduction” term suggested that many DEGs, which played a key role in T-2-induced apoptosis in pGCs, might be important RNA molecules affecting ovarian function in sows. These findings hinted that these DEGs might be in charge of controlling the T-2-induced apoptosis of pGCs. Meanwhile, our observations revealed the presence of 24 pathways that exhibited significant enrichment, including the PI3K-Akt signaling pathway, Ras signaling pathway, MAPK signaling pathway, apoptosis, and various crucial metabolic pathways. A variety of physiological or hazardous stimuli might activate the phosphatidylinositol 3-kinase (PI3K)-Akt signaling pathway, thereby controlling fundamental cellular processes such as apoptosis, transcription, survival, growth, proliferation, and translation. According to a previous study [31], DNA methylation as a result of the T-2 toxin regulates *RASSF4* expression and causes apoptosis through the downstream PI3K-Akt pathway. When rats were injured by SCI (spinal cord injury), activating the PI3K-Akt signaling pathway reduced caspase-9 and -3 expression levels and prevented apoptosis. Theoretically, it was believed that the PI3K-Akt signaling pathway also facilitates autophagy. The downstream kinase of the PI3K-Akt signaling pathway, mTOR, is closely related to autophagy. The PI3K/Akt/mTOR signaling pathway was involved in cellular autophagy in chicken hepatocytes and rat granulosa cells with polycystic ovary syndrome, respectively. Mitogen-activated protein kinases (MAPKs) are protein kinases that can be gradually phosphorylated to form a series of MAPK signaling pathways under stimulation by the environment, cytokines, and other stimuli, which play a significant role in regulating various biological processes such as cell migration, proliferation, and apoptosis [32]. A previous study has revealed that deletion of *Ksr1* in B cells (primary mice, 4~6 weeks old) resulted in inhibition of the Ras-MAPK signaling pathway; ultimately, B-cells exhibited decreased proliferation, increased apoptosis, and significantly delayed lymphomagensis [33]. Reddy et al. [34] found that Lanatoside C blocked the G2/M phase of the cell cycle by inhibiting the MAPK/Wnt/PAM signaling pathways, which was how it combated cancer. Male germ cell death was caused by the activation of the JNK/p38 MAPK pathway by T-2 mycotoxin [35]. Furthermore, *Talin1* improved endometrial cell adhesion by improving Ras signaling, resulting in easier embryo implanting [36]. As a result of ROS-mediated activation of intrinsic apoptosis, T-2 induced the apoptosis signaling pathway in rat granulosa cells.

Based on GSEA results, apoptosis and cell cycle abnormalities in pGCs with T-2 toxin exposure may be associated with the activation/inhibition of multiple important cell biological pathways. Members belonging to the Notch signaling pathway are differentially expressed in testicular somatic cells and germ cells, which are involved in the development of germ cells. Studies have shown that the Notch signaling pathway promotes the gonocyte’s apoptosis by positively regulating the activation of the pro-apoptotic genes *TRP53* and *TRP63* [37], and it was also found that the activation of Notch1 signaling can promote the apoptosis of spermatogonia in male mice [38]. In addition, the application of DAPT, a γ-secretase inhibitor, to cultured neo-natal ovaries leads to the disruption of Notch signaling, resulting in the retention of germ cells in nests and a decrease in the quantity of primordial follicles [39]. The NF-kappa B signaling pathway responded to the outside cell stimuli, which were associated with inflammatory responses, immune responses, cell growth, and other processes [40]. An in vitro study showed that NF-kappa B signaling pathway inactivation could reduce chondrocyte apoptosis and ECM degradation caused by T-2 toxin. The activation of the NF-kappa B signaling pathway has been reported to directly or indirectly mediate the cell cycle transition regulators’ expression to induce cell cycle arrest at G2/M and G0/G1 [41]. The Hippo pathway promotes apoptosis and inhibits cell proliferation by activating kinase cascades and suppressing the expression levels of effector molecules. Further studies found that interfering with the expression of *SAV1*, an upstream kinase gene of the Hippo pathway, induced the up-regulation of *GDF9* and *FSHR* gene expression in chicken preantral follicles, while the proliferation of chicken preantral follicular granulosa cells was significantly increased [42]. Plewes et al. [43] found that knocking out *YAP1* could inhibit E2, which indicated that *YAP1* may promote granulosa cell proliferation, support ovary function, and provide Hippo pathway inhibition hints. Interestingly, a large number of former investigations have suggested that inhibition/activation in cell signaling pathways are sometimes not always unilateral, of which extensive cross-talk keeps cells in certain physiological and pathological conditions. The constitutive activation of STAT3 and the NF-kappa B signaling pathway led to the up-regulation of Notch pathway promoter expression in human glioma CSC, which determined a new therapeutic target for glioma therapy [44]. Moreover, past research has shown that activated the Notch signaling pathway can repress specific promoter genes *Star*, *HSD3B2,* and *Cyp19a1* in the steroid biosynthesis pathway, which reveals that the steroid biosynthesis pathway that regulates follicular growth and atresia is negatively regulated by the Notch signaling pathway [45]. Taken together, all these previous studies in diverse cell types and our results in pGCs highlight a common mechanism for T-2 toxicity via induction of apoptosis.

## 5. Conclusions

In summary, our results demonstrated that the T-2 toxin causes adverse morphological changes, promotes apoptosis, and blocks cell cycle progression in pGCs. Transcriptional and bioinformatics analyses identified gene expression differences and molecular mechanisms in pGCs after T-2 toxin exposure, which suggested that the toxic effects of T-2 might be involved in biological processes such as cell damage, cell cycle regulation, and steroid metabolism through specific signaling pathways. Our findings offer a thorough understanding of the regulatory processes behind T-2 toxicity in swine ovarian granulosa cells.

## Figures and Tables

**Table 1:** Primer sequences used in qPCR experiments.

Gene Name	Primer Sequence (5′-3′)	Up/Down
*ATM*	CCTGCAAACCTTCATGTCCTAC	Down
	TTCTCCATTCCCGTTTCAACTG	
*CCNE2*	AGGAATTGTTGGCCACCTGTA	Down
	CAAACATCCTGTGAGCATCCC	
*SOS1*	GCAGAGGAACTGGCATTTGAC	Down
	AAATAAAGTGCCGCTCCAGG	
*SORT1*	TGGGGCAGGAGCAGTTCTA	Down
	TGGTGCCAAATCCGGTATCT	
*MRAS*	ATGAGTGACGGGGATGTTGA	Down
	GAAGGCTTTGTCCACGTTGA	
*AKT3*	AGGACCGCACACGTTTCTATG	Down
	CAACTTGAGATCCCGGTACACA	
*BAD*	CGGAGGATGAGTGACGAGTT	Up
	TTCCGGTACCACCAGGACT	
*MAP3K4*	GTGGAGAATGCGGAGGAATAC	Up
	TGAAGGAGCACTGCACGTTTT	
*BAX*	AGCTGCAGAGGATGATCGC	Up
	CCAGTTGAAGTTGCCGTCAG	
*PDPK1*	TGTGGGAGAACCTGCATCAT	Up
	TGGCTCAGGAGGTTGTCGTA	
*RELA*	CTGCCCCTAAAACCAACCAG	Up
	CACGGTCGGGTCAGTGTTAT	
*GADD45B*	GTGTCAGGAATGCAGCGACT	Up
	GGCTTTTCCAGGCATCTGTG	
*GAPDH*	ATTCCACCCACGGCAAGTT	*GAPDH-F*
	TTTGATGTTGGCGGGATCT	*GAPDH-R*

## Data Availability

The NCBI SRA database contains all read data (project ID: SRP254496).

## Supplementary Materials

The following supporting information can be downloaded at: https://www.mdpi.com/article/10.3390/genes15050579/s1, Table S1: RNA extraction test results; Table S2: RNA-seq data quality analysis results; Table S3: Differentially expressed genes of T-2 0 nmol/L and T-2 60 nmol/L in pGCs; Table S4: Significant GO terms enriched from DEGs; Table S5: Significant pathways enriched from DEGs; Figure S1: Representative results of GSEA plots depicting signaling pathways in T-2-treated pGCs compared to controls.
