# Supplementary material for: Mechanism of Apoptosis in Porcine Ovarian Granulosa Cells Triggered by T-2 Toxin
Source: Genes (Basel). 2024 May 1;15(5):579. doi: 10.3390/genes15050579 (PMC11120908; doi:10.3390/genes15050579)
Supplement: Supplementary file 1 [file genes-15-00579-s001.zip › Figure S1.pdf]

A

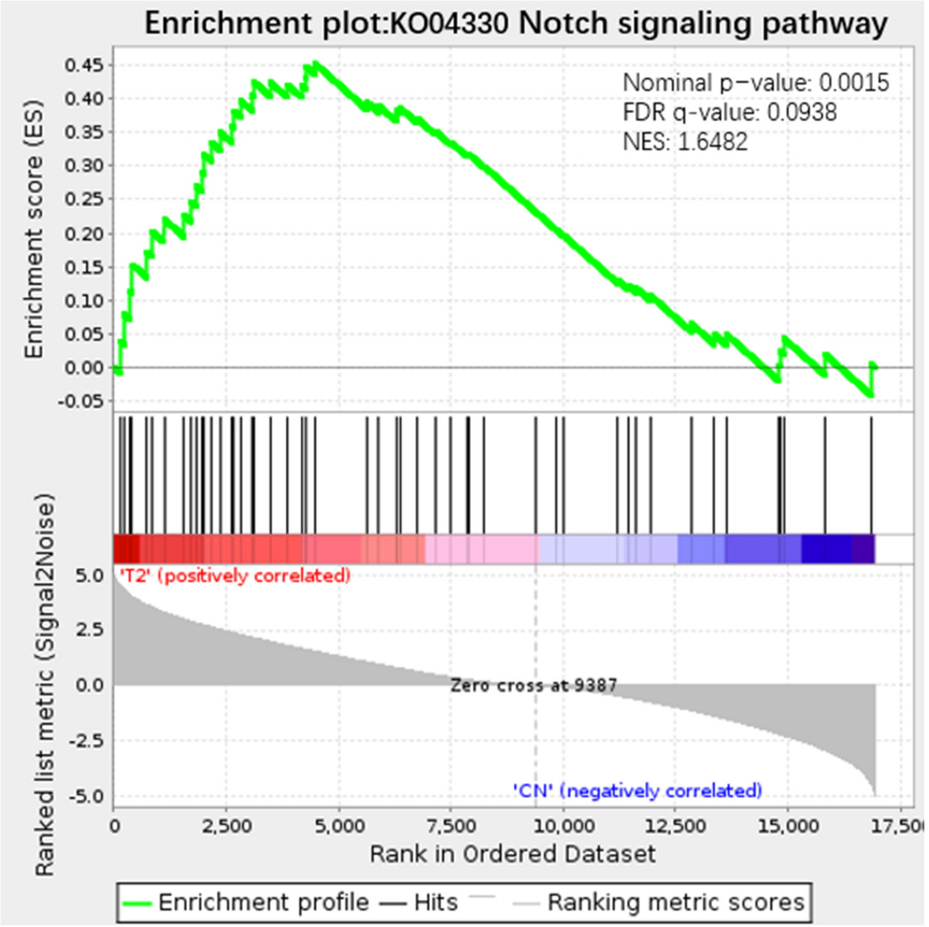

Enrichment plot:KO04330 Notch signaling pathway
Nominal p−value: 0.0015
FDR q-value: 0.0938
NES: 1.6482
Enrichment score (ES)
0.45
0.40
0.35
0.30
0.25
0.20
0.15
0.10
0.05
0.00
-0.05
'T2' (positively correlated)
Zero cross at 9387
'CN' (negatively correlated)
Ranked list metric (Signal2Noise)
5.0
2.5
0.0
-2.5
-5.0
0
2,500
5,000
7,500
10,000
12,500
15,000
Rank in Ordered Dataset
Enrichment profile
Hits
Ranking metric scores

B

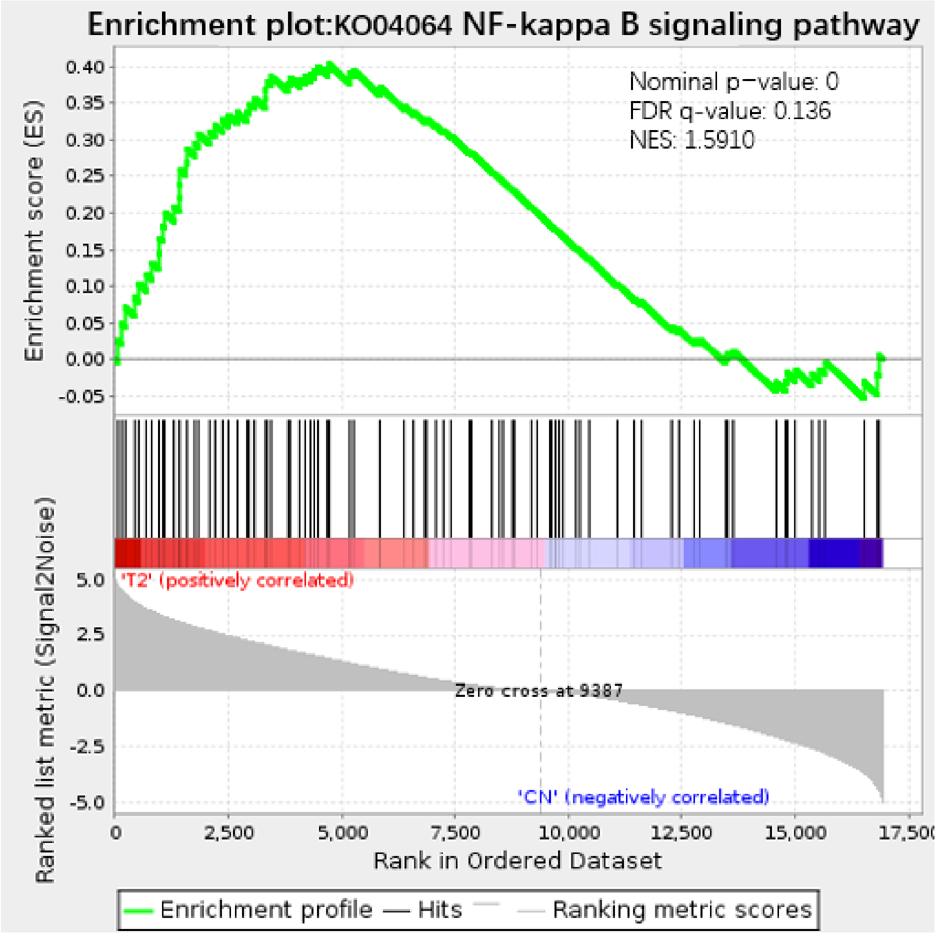

Enrichment plot:KO04064 NF-kappa B signaling pathway
Nominal p−value: 0
FDR q-value: 0.136
NES: 1.5910
Enrichment score (ES)
0.40
0.35
0.30
0.25
0.20
0.15
0.10
0.05
0.00
-0.05
'T2' (positively correlated)
Zero cross at 9387
'CN' (negatively correlated)
Ranked list metric (Signal2Noise)
5.0
2.5
0.0
-2.5
-5.0
0
2,500
5,000
7,500
10,000
12,500
15,000
Rank in Ordered Dataset
Enrichment profile
Hits
Ranking metric scores

C

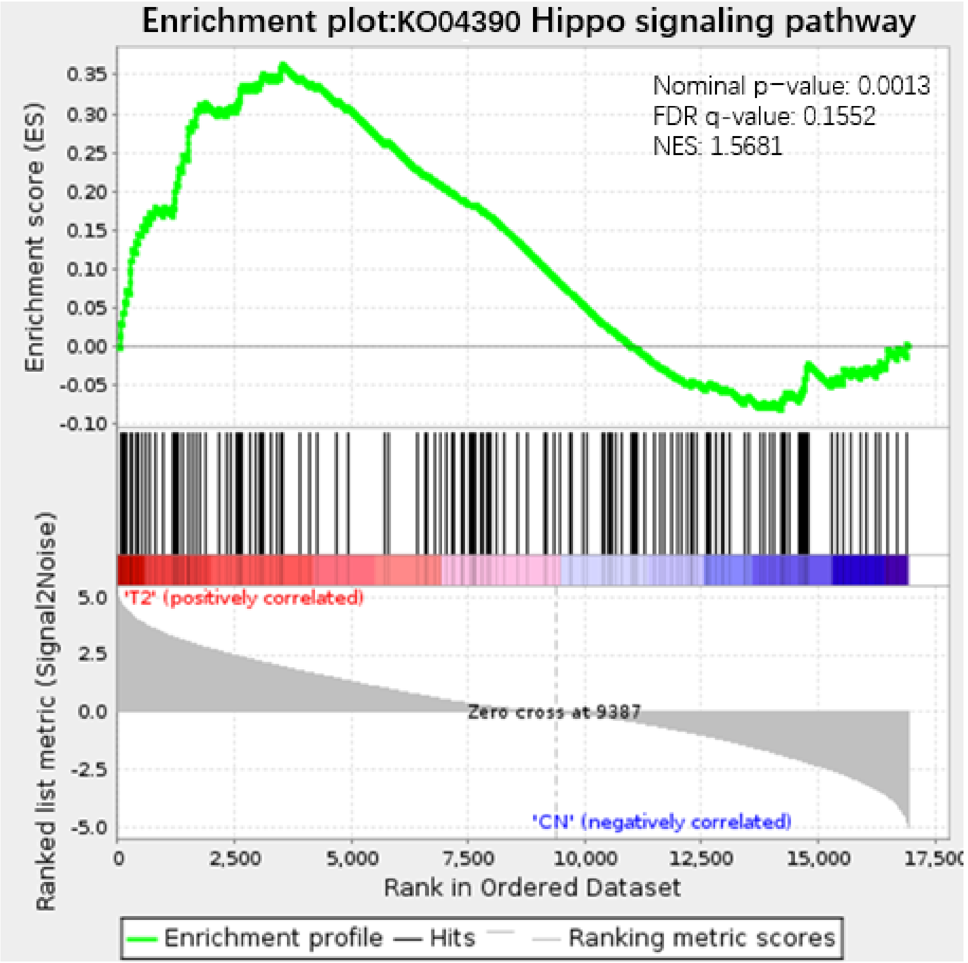

Enrichment plot:KO04390 Hippo signaling pathway
Nominal p−value: 0.0013
FDR q-value: 0.1552
NES: 1.5681
Enrichment score (ES)
0.35
0.30
0.25
0.20
0.15
0.10
0.05
0.00
-0.05
-0.10
'T2' (positively correlated)
Zero cross at 9387
'CN' (negatively correlated)
Ranked list metric (Signal2Noise)
5.0
2.5
0.0
-2.5
-5.0
0
2,500
5,000
7,500
10,000
12,500
15,000
17,500
Rank in Ordered Dataset
Enrichment profile
Hits
Ranking metric scores

D

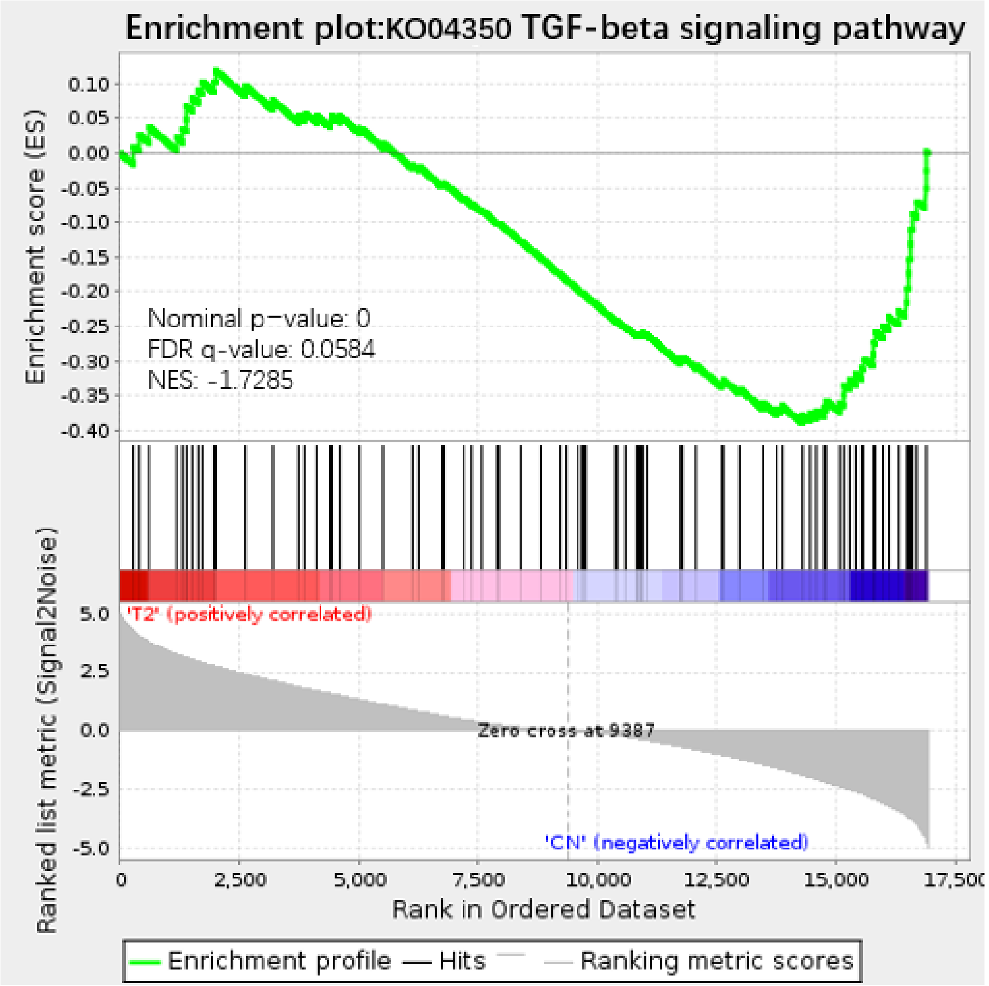
Enrichment plot:KO04350 TGF-beta signaling pathway
Enrichment score (ES)
0.10
0.05
0.00
-0.05
-0.10
-0.15
-0.20
-0.25
-0.30
-0.35
-0.40
Nominal p-value: 0
FDR q-value: 0.0584
NES: -1.7285
'T2' (positively correlated)
Ranked list metric (Signal2Noise)
5.0
2.5
0.0
-2.5
-5.0
Zero cross at 9387
'CN' (negatively correlated)
0
2,500
5,000
7,500
10,000
12,500
15,000
17,500
Rank in Ordered Dataset
Enrichment profile — Hits — Ranking metric scores

E

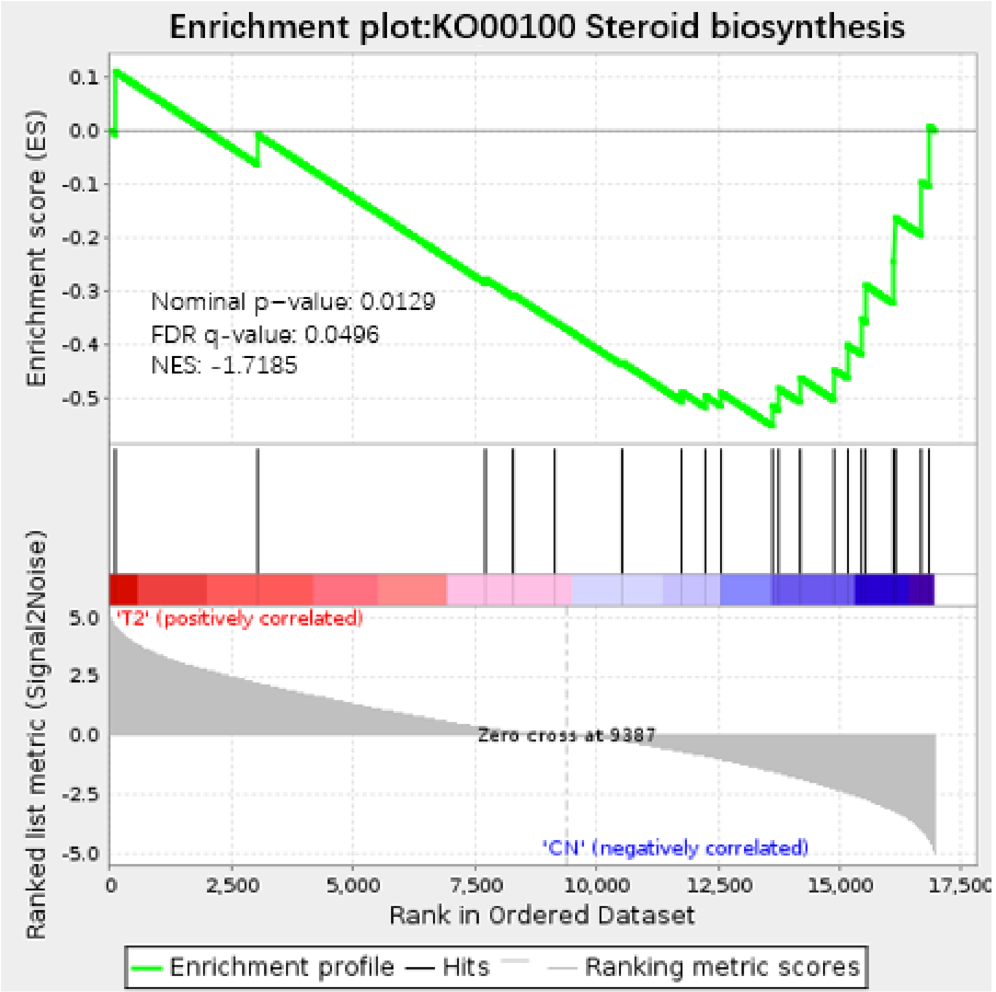

Enrichment plot:KO00100 Steroid biosynthesis
Enrichment score (ES)
0.1
0.0
-0.1
-0.2
-0.3
-0.4
-0.5
Nominal p−value: 0.0129
FDR q-value: 0.0496
NES: -1.7185
Ranked list metric (Signal2Noise)
5.0
2.5
0.0
-2.5
-5.0
'T2' (positively correlated)
Zero cross at 9387
'CN' (negatively correlated)
0
2,500
5,000
7,500
10,000
12,500
15,000
17,500
Rank in Ordered Dataset
Enrichment profile
Hits
Ranking metric scores

Figure S1. Representative results of GSEA plots depicting signaling pathways in T-2-treated pGCs compared to controls. Significantly enriched KEGG pathways in **(A)**Notch signaling pathway, **(B)**NF-kappa B signaling pathway, **(C)**Hippo signaling pathway, **(D)**TGF-beta signaling pathway and **(E)**Steriod biosynthesis. NES stands for normalized enrichment score.
